# Robotic Ultrasonic Testing Technology for Aero-Engine Blades

**DOI:** 10.3390/s23073729

**Published:** 2023-04-04

**Authors:** Pengzhi Ma, Chunguang Xu, Dingguo Xiao

**Affiliations:** School of Mechanical Engineering, Beijing Institute of Technology, Beijing 100081, China; xucg@bit.edu.cn (C.X.); xiaodg@bit.edu.cn (D.X.)

**Keywords:** ultrasonic transducer, ultrasonic testing, robot assisted, engine blade

## Abstract

With the rapid development of the aerospace industry, the quality inspection of complex curved components, such as aero-engine blades, is becoming increasingly strict. In contrast with other NDT methods, ultrasonic testing is easier to automate, while offering higher accuracy and efficiency in thickness measuring. To solve the challenge of the automated NDT inspection of aero-engine blades, in this study, an ultrasonic inspection system with a six degree of freedom (DOF) was proposed for industrial robots. Additionally, a defect detection model and a thickness detection method were proposed for the robotic ultrasonic inspection system, based on the thickness variation of the aero-engine blade. Through the quantitative analysis on engine blades with prefabricated defects and curved test blocks with stepped thicknesses, it can be concluded that our system is able to achieve high accuracy in defect detection and thickness measurement.

## 1. Introduction

The blade, as a rotating component, is of great importance to an aero-engine. Used in harsh environments, it is subjected to complex loads, thereby it is prone to fatigue defects due to excessive stress. Blade defects are an important factor affecting flight safety as even minor defects can cause serious consequences to the aircraft, endangering the safety of the flight [1]. To ensure the proper working condition of the blade and reduce the air holes, inclusions, debonding, and other defects caused in the production process, such as the fatigue defects generated through use, as well as other impacts on the service life of the blade, the non-destructive testing of aero-engine blades is used as an effective means to reduce huge economic costs.

Nowadays, ultrasonic inspection, eddy current inspection, penetration inspection, and radiographic inspection are commonly applied to detect blades. Among them, eddy current inspection is more sensitive to surface and near-surface defects, but it is difficult to achieve internal defect detection. For penetration detection, the blade should be coated before detection, which is complex and costly, and it is also hard to identify internal crack defects in the blade. The radiographic inspection method is limited by the transmission method; it is of poor display clarity and is unable to effectively characterize internal crack defects. Ultrasonic inspection technology is commonly used in the thickness measurement and defect detection of aero-engine blades, and its use of ultrasonic leaky Rayleigh waves can achieve the detection of surface and subsurface crack defects [2,3]. Near surface defects are difficult to identify in the near surface blind zone because the defect echo always overlaps with the interface echo. Based on the combination of signal feature extraction and GA-SVM, near-surface defects can be quantitatively detected [4]. The probabilities of ultrasonic testing can be calculated as a function of defect size (POD curve) at different defect depths, which were generated based on the Monte Carlo simulation [5,6].

The traditional ultrasonic non-destructive testing equipment cannot realize automated inspection of complex curved components, especially the high-precision profiling of the ones with significant variation in curvature, thickness, and shape. By contrast, industrial robotic ultrasonic testing systems can efficiently achieve blade defect detection and thickness measurement.

In recent years, plenty of research has been conducted on robotic NDT technology and its related equipment. Xiao [7] calibrated a robot using an ultrasonic alignment method on the basis of a quaternion algorithm to meet the constraint requirement for the ultrasonic incidence angle, achieving a higher accuracy of the robot trajectory compared with other NDT methods. Secanellas [8] developed a stand-alone synchronization system based on the calculation of post-calibration experiment synchronization mapping and the alignment of the positive mapping, enabling industrial robots to generate high-quality ultrasonic images of the components with complex geometric shapes. Zhang [9] proposed a robot-assisted wheel crack extension assessment model to quantitatively analyze wheels with a series of longitudinal and transverse pre-cracks. In addition, more research on robotic NDT systems has been carried out by Carmelo Mineo’s team in the UK [10,11,12,13,14]. Mineo and Cooper [10] developed a dual-robot ultrasonic NDT system that reduced inspection time by a factor of four for complex geometric surfaces and composite components. Each robot in this system can work independently or collaboratively, allowing coordinated movements to scan one area of the component by ultrasonic penetration, or a single robot to inspect two different areas of the component simultaneously by ultrasonic reflection. For the non-destructive inspection of complex curved workpieces, Mineo [11] investigated the trajectory planning of a non-destructive inspection robot and subsequently developed a trajectory planning software specifically for robotic non-destructive inspection systems based on the MATLAB toolbox [13]. He also designed a special instrument called FIToolbox for high-speed analog data acquisition, so as to improve the data acquisition speed and inspection speed of the robotic inspection system [14]. Guo [15] used a robotic NDT system to achieve the automated ultrasonic NDT of semi-enclosed components. Liu [16] proposed a six-point positioning scheme to update the tool coordinate system quickly and accurately, and realized the ultrasonic measurement of engine blade thickness by industrial robots.

This paper presents an industrial robot-based ultrasonic non-destructive inspection technique that allows for the non-destructive assessment of aero-engine blade defects and thicknesses. We propose an ultrasonic inspection method applicable to the regions of the blade body and the inlet and exhaust sides with high accuracy in thickness measurement. The performance of the robotic ultrasonic inspection system in defect detection and thickness measurement was verified through the inspection of blades with prefabricated defects and stepped thicknesses.

## 2. Experimental Methods and Configuration

### 2.1. Thickness Measurement

Following the principle of ultrasonic inspection, the accuracy of the robotic sweeping trajectory is required for the system to ensure a constant hydroacoustic range. The use of ultrasonic transducers with narrow bandwidth, small wavelength, and high frequency can guarantee that the oscillation period of the ultrasonic head wave is shorter than the propagation time of the ultrasonic wave in the blade, which improves the accuracy of thickness detection.

**Figure 1:**
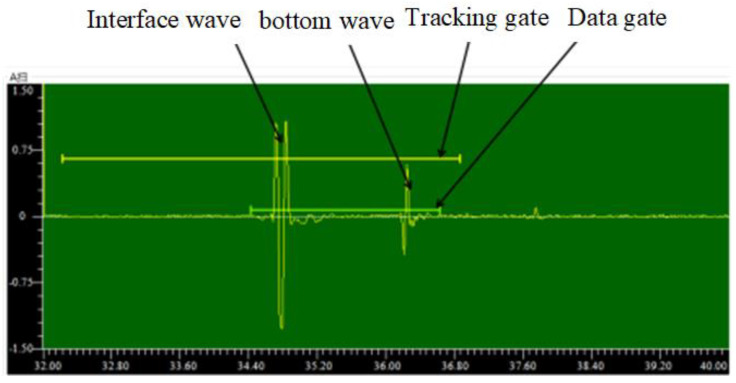
Schematic diagram of the interface reflection echo gate tracking.

The analysis of the time-domain sampling signal using the time-of-flight (TOF) method has a serious impact on the accuracy of thickness measurement [17]. The gate tracking technique can be applied to identify the time-domain positions of the reflected echoes from the interface, as shown in Figure 1. For the thickness measurement, the selection of the ultrasonic waveform data containing the head and bottom waves is required for autocorrelation analysis to accurately derive the acoustic time difference.

In the actual detection system, the original signal is acquired, and the noise is reduced and interpolated to obtain an ultrasonic echo signal *x*(*m*) with a length of 2N. After this step, the method for the calculation of blade thickness is as follows:

(1) Before the actual inspection, the actual thickness of the specimen at the easy-to-measure location should be measured using standard equipment, such as a CMM. Then, the actual ultrasonic propagation velocity C_U_ in the material under test should be calibrated at the measurement point with a known thickness;

(2) The first N consecutive waveform data points from the first data point in *x*(*m*) are taken as the matching signal *y*(*n*), and the length of the data acquisition gate must essentially cover the time domain range in which the head and bottom waves are included;

(3) The N of these consecutive waveform data in *x*(*m*)are taken and recomposed into a new signal *zi*(*n*), where *i* denotes the first data point position of this waveform data, which satisfies 0 ≤ *i* ≤ N − 1;

(4) The discrete autocorrelation function *r*(*i*) is computed for *y*(*n*) and *zi*(*n*)*;*

(1) $$r(i)=\sum_{n=0}^{N-1}y(n)z_{i}(n)$$

(5) Various values of *i* are taken in the range [0, N − 1] and step (3) is repeated for all *r*(*i*);

(6) The extreme value of *r*(*i*) is identified for a distance from the origin (*i* = 0) and then the corresponding data point location *i* is determined by appropriately rounding off the value of *r*(*i*) near the origin (*i* = 0) based on the time-domain pulse width;

(7) The time at which the autocorrelation function *r*(*i*) reaches its peak is calculated from the value of *i*, combined with the sampling frequency *f* and the interpolation multiplier *n*. The acoustic time difference Δ*t* between the ultrasonic head wave and the bottom wave is also determined as follows:

(2) $$\Delta t=\frac{i\cdot n}{f}$$

(8) The propagation velocity *C_U_* of the ultrasound waves in the measured material and the acoustic time difference Δ*t* are used to calculate the actual thickness *T* at each discrete sampling point.

(3) $$T=\frac{C_{U}\cdot \Delta t}{2}$$

**Figure 2:**
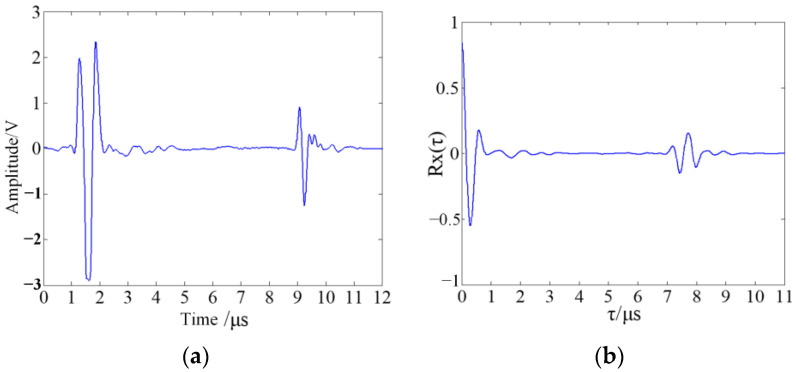
Ultrasonic echo signal graph and autocorrelation function curve for autocorrelation analysis. (**a**) Ultrasonic echo signal graph. (**b**) Autocorrelation function curve.

In the actual inspection process, the collected ultrasonic echo signal for autocorrelation analysis and its corresponding autocorrelation function curve are given in Figure 2, which shows that in the autocorrelation function curve, there is a great value appearing far away from the origin.

The location where the autocorrelation function achieves its maximum value reflects the time-domain interval of the two ultrasound signal segments with the highest similarity.

### 2.2. Inlet and Exhaust Side Defect Detection Method

**Figure 3:**
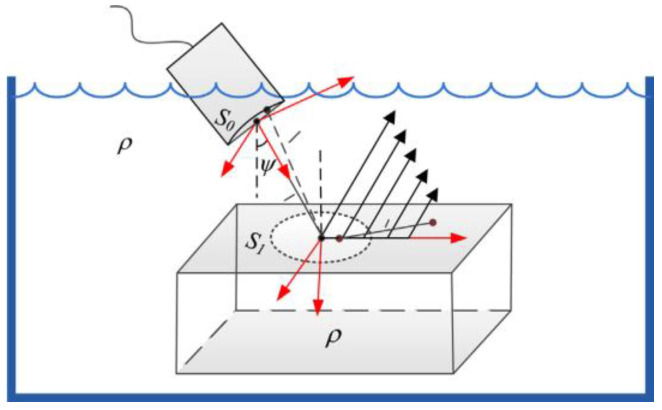
Schematic diagram of the LRW generation and the definition of the coordinate systems [18].

Ultrasonic inspection with the use of leaky Rayleigh waves is effective for the measurement of micro defects existing on surfaces. Waveform conversion occurs when a longitudinal wave enters the surface at a specific incident angle, as shown in Figure 3, following Snell’s law. The leaky Rayleigh wave is generated by the critical refraction of the longitudinal wave from an immersion transducer with a second critical angle. Beyond the second critical angle, the transmitted shear wave becomes a leaky surface wave as well.

Li [18] proposed a non-near-axis multi-Gaussian beam (NMGB) model for leaky Rayleigh waves (LRW) generated by a focused submerged transducer with tilted incidence and also verified the quantitative relationship between the curvature of different materials and the attenuation due to leakage. Li [19] proposed an imaging detection method that combines leaky Rayleigh wave detection and synthetic aperture focusing technique (SAFT) with immersion pulse-echo scanning. By incorporating the attenuation of LRW during planar propagation, the NMGB model of the LRW velocity field can be expressed as:

(4) $$\begin{array}{c} v\left(x_{A_{1}},y_{A_{1}}\right)=\frac{T\omega \rho_{f}\pi }{2Q}\frac{\exp(i\pi /4)}{\sqrt{2\pi k_{r}}}\sum_{n=1}^{25}\frac{A_{n}\exp\left(ik_{f}r_{10}+ik_{r}R_{2}-\alpha_{f}r_{10}-\alpha_{LRW}x_{A_{1}}\right)}{\sqrt{M_{111}M_{222}R_{2}}\left(1+iB_{n}r_{10}/D\right)}\\ \times \exp\left(-\frac{i}{2}\frac{{\left(k_{f}\sin\psi -k_{r}x_{A_{1}}/R_{2}\right)}^{2}}{M_{111}}\right)\exp\left(-\frac{i}{2}\frac{{\left(k_{r}y_{A_{1}}/R_{2}\right)}^{2}}{M_{222}}\right) \end{array}$$

In accordance with Equation (4), one of the main results of Li’s study, it can be seen that the velocity field mainly depends on the water range, the parameters of the focusing sensor, and the attenuation of the LRW.

**Figure 4:**
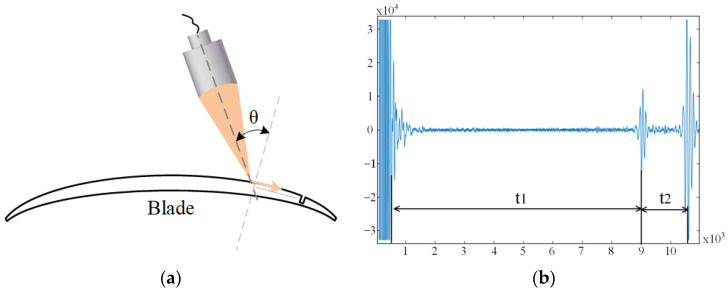
Leaky Rayleigh waves are generated by oblique incidence of the transducer. (**a**) Oblique incidence of the transducer. (**b**) Leaky Rayleigh wave chart.

It can be observed that the leakage of Rayleigh waves on the surface of the specimen encounters a small interface where the acoustic impedance changes, and at this interface, a reflected wave is generated and propagates in the opposite direction to the original propagation. Figure 4 shows a typical detection A-scan waveform of the leaky Rayleigh wave: after a certain water distance (t1), the emission pulse, due to the different surface roughness of the specimen, and the probe can receive the interface scattering waves directly scattered from the specimen surface. When the probe is swept close to surface and subsurface defects, a defect echo appears after the interface scattering wave. The position of the defect echo varies with the distance between the probe and the defect, and the time difference between this signal and the interfacial scattering wave (t2) is the time for the leaky Rayleigh wave to propagate back and forth across the surface. Therefore, we chose to capture the signal of the leaky Rayleigh wave in the ultrasonic B-scan, which must be time-gated immediately after the interface scattering wave.

### 2.3. System Components

**Figure 5:**
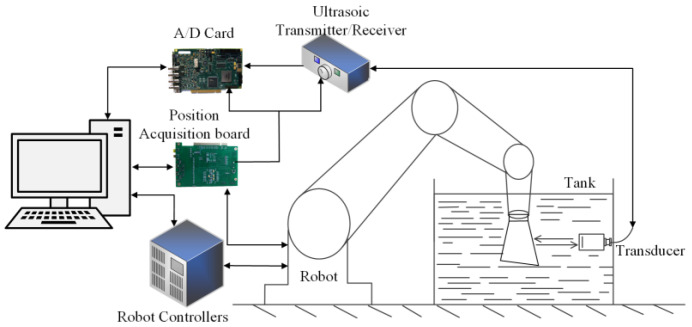
Overview of the robot-assisted UT system.

The six-degree-of-freedom industrial robot with high flexibility can effectively achieve multi-angle incidence of the acoustic beam on complex surfaces. The ultrasonic inspection system of the industrial robot developed in this study is shown in Figure 5.

Compared with the conventional method of robotic ultrasonic probe gripping (UPGR), the TOGR solution consumes less time in the inspection of complex specimens when the blade is mounted on the end-effector of a Staubli industrial robot [20]. It shows a significant improvement in the position accuracy of the robot trajectory. The computer generates the scan path offline according to the part of the blade being inspected and the specific inspection method. During the sweeping process, the robot sends synchronized signals to the pulse transceiver according to the sweeping trajectory, enabling the simultaneous acquisition of the spatial position of the robot end-effector and the ultrasound signal. In the developed software for robotic ultrasonic scanning and imaging, the ultrasonic signal data is combined with the corresponding robot posture data to carry out multiple types of ultrasonic imaging and thickness measurement.

### 2.4. Robot Sweep Track Planning

**Figure 6:**
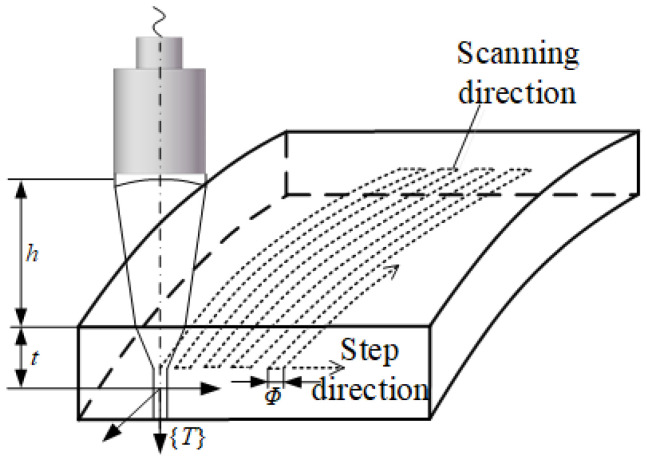
Ultrasonic transducer surface sweep path.

The robot sweep path is based on CAD/CAM for trajectory planning. Xiao Zhen and C. Melo provided a detailed introduction to robot trajectory planning and proposed a variety of techniques on trajectory planning and calibration. When developing the blade sweeping trajectory according to the inspection task, it is necessary to take into account the focal length of the ultrasonic energizer F, the focal column diameter Φ, the material sound velocity CM, and the sound velocity in water CW, so as to obtain the optimal detection sensitivity and resolving power. Taking the path planning of the focusing probe for surface detection as an example, as shown in Figure 6, the distance between the transducer surface and the incident surface is the water distance h, which is calculated as:

(5) $$h=F-t\left(C_{M}/C_{W}\right)$$

In Equation (5), t represents the depth of focus, and the water range can be calculated from Equation (5) according to the requirements of the detection depth so that the focus is located at the detection depth. For path planning of the surface detection shown in Figure 6, the tool coordinate system {T} is established at the focal point, the water range is kept as h, the step interval is approximated as half the diameter of the acoustic beam, and the incidence angle is kept coincident with the surface point normal or at a certain angle to achieve better coverage of the entire detection area.

**Figure 7:**
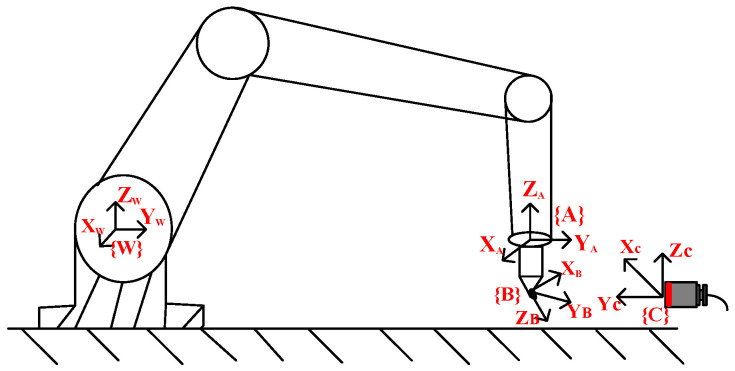
Coordinate system distribution of the robotic inspection system.

The coordinate system distribution of the robotic NDT system is shown in Figure 7. {A} denotes the tool coordinate system of the end flange of the robot, {B} denotes the workpiece coordinate system of the blade, {C} denotes the user coordinate system of the location of the ultrasonic transducer, and {W} denotes the reference coordinate system of the robot. In accordance with the robot kinematics theory, as long as the D-H parameters of the manipulator are determined, the relative locations of the tool coordinate system {A} and the reference coordinate system {W} can be deduced.

To obtain a stable ultrasonic pulse echo signal, the ultrasonic transducer beam axis should be aligned with the surface normal at the sweep point of the blade under test or a specific deflection angle. The normal vectors of the sweep point with respect to the coordinate systems {A}, {B}, and {C} are denoted as AP, BP, and CP respectively. Taking the requirement for the incidence angle of ultrasonic longitudinal waves in the vertical re-flection method as an example, to ensure that the normal direction of the discrete point BP is consistent with the beam axis CP of the ultrasonic transducer, it is necessary to solve the locus attitude satisfied by {A} when the coordinate system {B} is rotated to {C}. When the auxiliary coordinate system is rotated from {B} to {C}, we obtain

(6) PA=TBATCBPC=TCAPC

The positional transformation matrix TCA of the coordinate systems {A} and {C} can be decomposed into a rotation matrix RCA and the position translation vector PAORGC in the form of

(7) TAC=[RACPAORGC01]=[RTBCRTABRBCPAORGB+PBORGC01]=[RTBCRTAB−RBCRTBAPBORGA+PBORGC01]

When the machining point data from the CAD/CAM post-processing file is obtained, the position of the coordinate system {B} with respect to {A} can be expressed as the transformation matrix RBA:

(8) RBAT=PAP−1B=[ξXφXψXξYφYψYξZφZψZ]

where $\xi ={\left[\xi_{X},\xi_{Y},\xi_{Z}\right]}^{T}$, $\varphi ={\left[\varphi_{X},\varphi_{Y},\varphi_{Z}\right]}^{T}$, and $\psi ={\left[\psi_{X},\psi_{Y},\psi_{Z}\right]}^{T}$ represent the unit vectors of the x-, y-, and *z*-axis directions of {B} in {A}, respectively, which are related to the tangential vector, normal vector, and the fork product of the tangential vector and the normal vector, respectively, at that point.

When the ultrasonic transducer beam axis coincides with the normal of the planned discrete point of the measured part, i.e., the coordinate system {B}, coincides exactly with the *Y*-axis of {C} (the rotation matrix RCB is the unit matrix) or is inversely co-linear in the *Y*-axis vector direction, the rotation matrix RCB can be calculated as

(9) RCBPC=PB

When {B} coincides with {C}, RCB can be defined as the unit matrix.

(10) RCB=[100010001]

The extrapolated TAC is

(11) TAC==[RTAB−RABPBORGA+PBORGC01]

**Figure 8:**
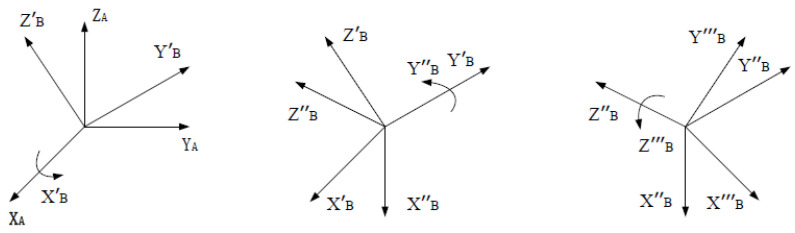
Rotation matrix transformation order.

The TX90L manipulator used in the system is in accordance with the transformation order of the X-Y-Z Euler angles rotation matrix, as shown in Figure 8. In other words, the coordinate systems {X_A_, Y_A_, Z_A_ } are rotated by the α, β, and γ angles around the X, Y, and Z axes in the last rotational attitude to get the coordinate systems {$X_{B}^{\prime \prime \prime }$,$Y_{B}^{\prime \prime \prime }$,$Z_{B}^{\prime \prime \prime }$}. Therefore, only the Euler angles between the trajectory points should be solved to comprehend their positional transformation.

The transformation matrix required to rotate the coordinate system from the locus {A} to {C} is

(12) $$\begin{array}{ll} R_{X-Y-Z} & =R_{X}(\alpha )R_{Y}(\beta )R_{Z}(\gamma )=\left[\begin{array}{ccc} 1 & 0 & 0\\ 0 & \cos\alpha & -\sin\alpha \\ 0 & \sin\alpha & \cos\alpha \end{array}\right]\left[\begin{array}{ccc} \cos\beta & 0 & \sin\beta \\ 0 & 1 & 0\\ -\sin\beta & 0 & \cos\beta \end{array}\right]\left[\begin{array}{ccc} \cos\gamma & -\sin\gamma & 0\\ \sin\gamma & \cos\gamma & 0\\ 0 & 0 & 1 \end{array}\right]\\ & =\left[\begin{array}{ccc} \cos\beta \cos\gamma & -\cos\beta \sin\gamma & \sin\beta \\ \sin\alpha \sin\beta \cos\gamma +\cos\alpha \sin\gamma & -\sin\alpha \sin\beta \sin\gamma +\cos\alpha \cos\gamma & -\sin\alpha \cos\beta \\ -\cos\alpha \sin\beta \cos\gamma +\sin\alpha \sin\gamma & \cos\alpha \sin\beta \sin\gamma +\sin\alpha \cos\gamma & \cos\alpha \cos\beta \end{array}\right] \end{array}$$

Substituting the rotation matrix RTAB in Equation (11) into Equation (12), the Euler angles [α, β, γ] required to rotate the manipulator tool coordinate system {A} to {C} can be derived, which in turn solves the input parameters required for the manipulator to reach the specified discrete point poses.

(13) {α=atan2(−φxcosβ,ξxcosβ)β=atan2(−ψx,ξx2+φx2)γ=atan2(−ψycosβ,ψzcosβ)PAORGC=−RABPBORGA+PBORGC

where PAORGC is the position translation vector for translating {A} to {C}. The liquid immersion ultrasonic focusing transducer should maintain a constant acoustic range *d* with the measured surface, which is decomposed into three coordinate directional components, and the positional parameters input to the manipulator controller are [PAORGXC−dx,PAORGYC−dy,PAORGZC−dz,α,β,γ].

According to the type of defects and ultrasonic detection constraints, it is necessary to qualify the coordinate system poses of the discrete points, mainly to determine the deflection angle θ of the ultrasonic transducer beam axis relative to the normal vector of the measured surface. For example, θ is generally made to detect the internal defects of a workpiece, so that the ultrasonic beam is incident perpendicular to the surface, i.e., {B} and {C} ensure that the normal vectors are parallel or reversed, using the unit matrix shown in Equation (10) or the positive definite matrix with 180° rotation around the *Z*-axis shown in Equation (14) shown in the positive definite matrix rotated 180° around the *Z*-axis to solve:

(14) RCB=[1000−1000−1] or RCB=[−10001000−1]

### 2.5. Samples Preparation

**Figure 9:**
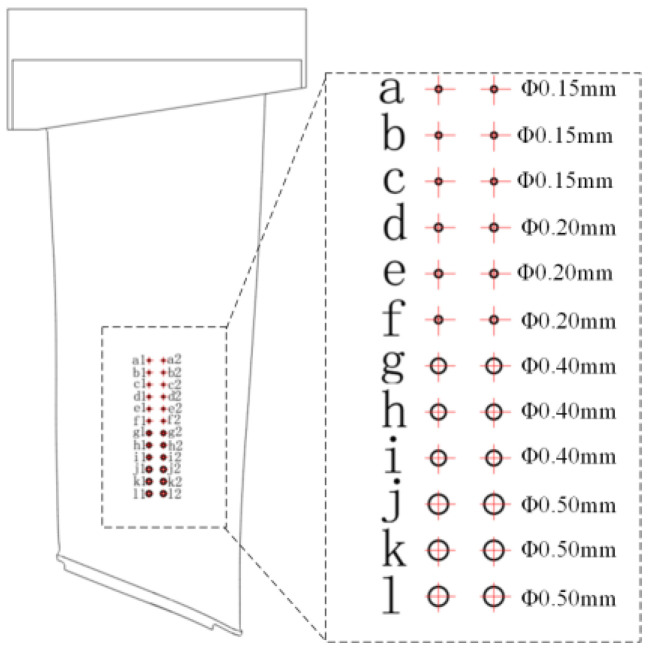
Artificial flat bottom hole and crack defects of blade body.

**Figure 10:**
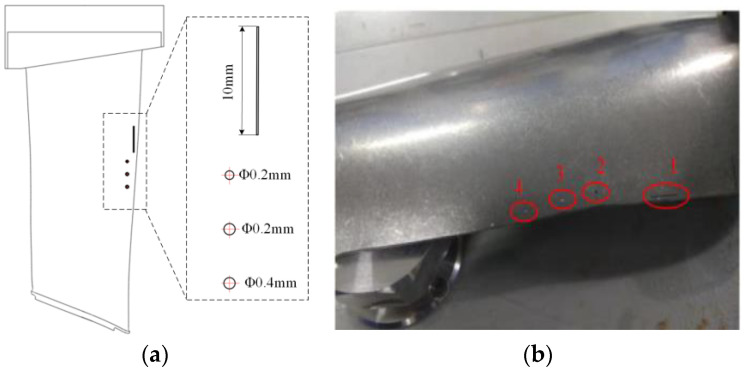
Artificial flat bottom hole and crack defects on air inlet side. (**a**) Prefabricated defect at blade edge. (**b**) Titanium engine blade.

**Figure 11:**
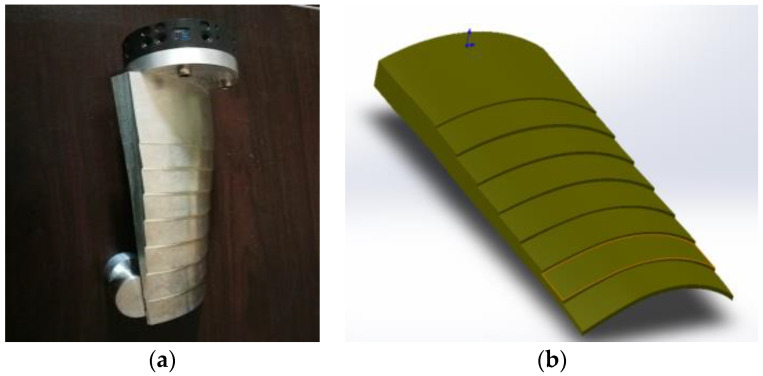
Simulated blade curved surface and stepped thickness component. (**a**) Simulated blade test block with curved surface for thickness measurement. (**b**) Designed model.

The main failure forms of aeronautical blades are fatigue, creep deformation, corrosion, oxidation, and surface degradation caused by overheating, and these forms of failure are concentrated at the blade body, inlet, and outlet edges. For example, corrosion occurs at the blade body, while cracks and defects are formed at the inlet and outlet edges, with the damage at the inlet edge being the most severe. To verify the defect detection performance of the robotic ultrasonic inspection system, a titanium blade with multiple defects of flat bottom holes and sub-millimeter cracks was selected, as shown in Figure 9 and Figure 10. To further verify the accuracy of the system in measuring blade thickness, we designed blade test blocks with stepped thicknesses based on the CAD model of the engine blade in the range of 2–12 mm, as shown in Figure 11.

## 3. Experiment Results and Discussion

**Figure 12:**
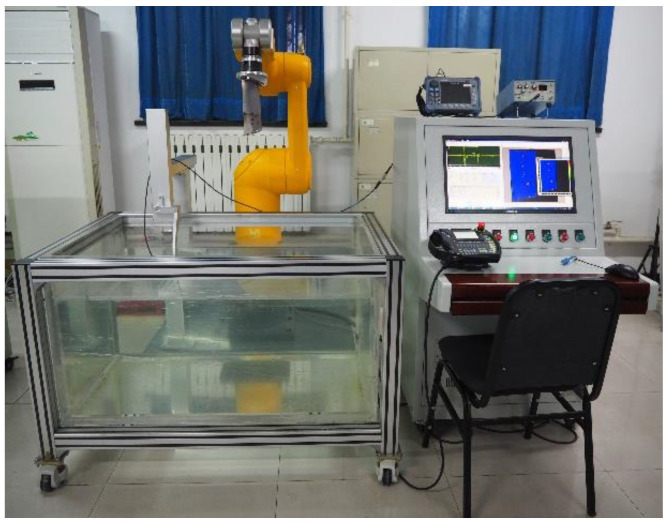
Scheme of ultrasonic nondestructive testing system for robotic.

In the present study, the system depicted in Figure 12 was designed to evaluate engine blades. It can be observed that the blade to be tested is mounted on the end-effector of a Stäubli industrial robot and it is moved relative to a fixed ultrasonic transducer for sweeping inspection. The details of the components are listed in Table 1.

### 3.1. Thickness Measurement

Based on the principle of vertical reflection of longitudinal pulse, the ultrasonic thickness measurement for aero-engine blades with complex curved workpieces of variable thicknesses adopts the sweeping method, with robot clamping at the blade. Firstly, noise reduction and interpolation are carried out on the ultrasonic A sweep waveform signals collected by the system. Then, the propagation time of ultrasonic waves in the blade under test is obtained by using autocorrelation function analysis combined with the sound velocity of the calibrated material to accurately calculate blade thickness. In addition, this method can achieve high measurement accuracy without a high requirement for the high structural characteristics of the test piece.

The method of ultrasonic detection applied to the simulated blade test block with a curved surface for thickness measurement is to randomly select two points on the CAD model with the same thickness value. These two points are calibrated and measured through utilizing a coordinate measuring machine (CMM). The sound velocity of the calibrated material is then used for detecting the thickness of other points, with the results shown in Table 2. It can be observed that the detection thickness error is less than ±0.02 mm.

### 3.2. B-Scan Imaging of the Exhaust Gas Edge

**Figure 13:**
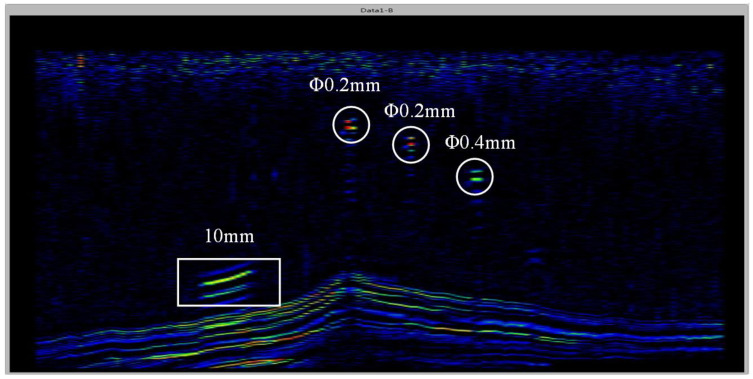
Ultrasonic B-scan images of inlet and exhaust edges of the blade.

For the prefabricated defects at the blade inlet and exhaust side, we use ultrasonic longitudinal waves with the incidence angle greater than the second critical angle to generate the leaky Rayleigh wave. The ultrasonic transducer sweeps with a fixed angle along the inlet and exhaust side and the ultrasonic B-scan imaging is used for detection. As shown in Figure 13, it can be seen that the results are in line with the sizes and locations of the prefabricated defects. Therefore, this method can be used to detect the flat bottom hole defects with a diameter of 0.2 mm and crack defects with a width of 0.2 mm at the inlet and exhaust side of the engine blade, meeting the surface defects’ detection requirements for the inlet and exhaust side of the engine blade.

### 3.3. C-Scan Imaging of the Leaf body

**Figure 14:**
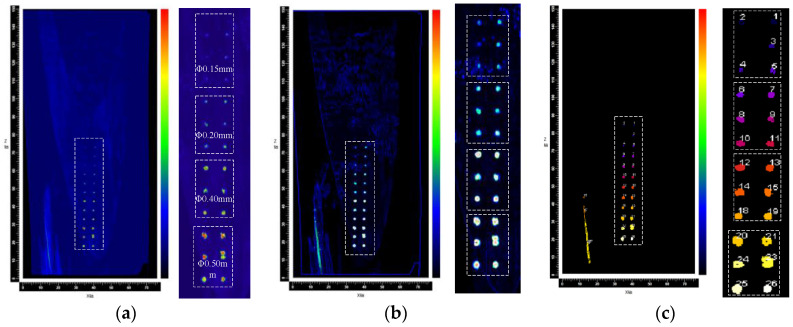
Ultrasonic C-scan images for defects with a diameter of 0.15 mm. (**a**) Original ultrasonic C-scan image. (**b**) Identification of defect contours for contrast. (**c**) Defect identification calibration.

Figure 14a shows the C-scan images of the ultrasonic vertical incidence detection of prefabricated flat bottom hole defects on the leaf body. By convolution of the original image, the defect feature can be highlighted, as shown in Figure 14b. Then, a reasonable gray threshold can be set to extract the prefabricated defect information, and finally realize the automatic identification and labeling of the prefabricated defect of the blade, as shown in Figure 14c. From the processed ultrasonic C-scan imaging, it can be seen that the distribution of defects is consistent with the position of prefabricated defects on the blade test block.

**Figure 15:**
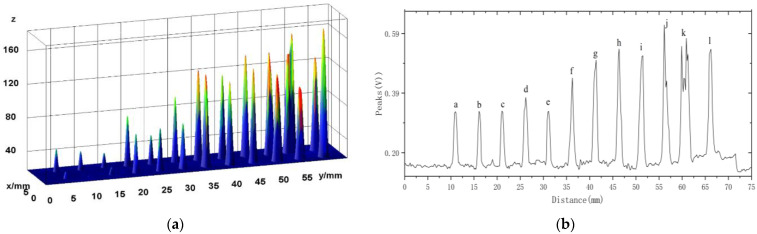
Flat bottom hole defect image intensity value. (**a**)original amplitude (**b**) amplitude curve normalized results. Lowercased letters correspond to Figure 9.

We extract the gray value of the defect in Figure 14a and convert the gray value of the image into signal amplitude; the obtained original amplitude curve and normalized results are shown in Figure 15. The figure shows that the difference between the echo and the noise signal amplitude strength of the smallest flat-bottomed hole defect with a diameter of 0.15 mm reaches −6 dB, which proves that the ultrasonic longitudinal wave vertical reflection method can effectively detect internal defects of the blade larger than this size. It can be seen from the curve that, in the case of the same depth, the larger the defect diameter of the flat-bottomed hole, the greater the maximum amplitude intensity of the echo signal, the wider the amplitude distribution range, and the larger the scanning imaging size of the defect. Therefore, the size of the defect can be compared with the variation range of the gray value in the C-scan image. Hence, the system can detect the standard flat bottom hole defect with 0.15 mm in diameter, meeting the detection requirements for quantitative analysis of defects.

## 4. Conclusions

In this study, a robot-based ultrasonic NDT system solution was proposed to solve the challenge of automated non-destructive inspection of aero-engine blades. The ultrasonic thickness measurement method based on the principle of longitudinal wave pulse vertical reflection was discussed, and the correlation calculation of the ultrasonic echo signal was carried out to obtain the accurate propagation time of the ultrasonic wave inside of the tested part, and the calculation of the thickness value at a specific point was realized. Based on the characteristics of leaky Rayleigh wave generation and propagation, a robot-assisted leaky Rayleigh wave B-scan imaging is proposed, which can effectively and quickly detect surface and subsurface defects of engine blades. The focus depth of the ultrasonic beam is analyzed when the robot scans the trajectory, and the mathematical model of the coordinate transformation between the robot reference coordinate system and the workpiece coordinate system is established, and the robot trajectory planning is realized through the algorithm of matrix transformation. In order to verify whether the proposed method meets the detection needs of engine blade thickness and tiny defects, we designed multiple sets of experiments to verify the accuracy of robot detection trajectory, ultrasonic scanning image detection of defects at different positions, and the tested samples of blade thickness. The results show that the system can effectively detect a hollow defect with a diameter of 0.15 mm with a thickness measurement error of no more than 0.02 mm. It has been verified that the robotic NDT system is suitable for the NDT inspection of aero-engine blades.

We used the engine blade as the main verification carrier of the test to verify the detection performance of the robot ultrasonic detection system. Based on this, the trajectory planning algorithm, thickness measurement method, subsurface and internal micro defect detection method, and ultrasonic scanning image representation of the defect characteristics and spatial distribution of the tested part proposed by us can also be applied to non-destructive testing and evaluation of other components with complex surfaces.

## Figures and Tables

**Table 1:** Main components of the robot-assisted UT system.

Apparatus	Brand	Model	Property
Robot	Staubli	TX90L	Repeatability: 0.035 mm
Ultrasonic Pulser/Reciver	Olympus	5077PR	Receiving bandwidth: 1 KHz–35 MHz
Ultrasonic probe	Olympus	V319	20 MHz
A/D Acquisition Card	Acquisition Logic	AL12200	sample rate: 250 MHz
Container	Non-standard	Non-standard	Volume: 500 L

**Table 2:** Test results of components with simulated blade thickness (mm).

Standard Value	CMM Calibration	Measurement Results	Measurement Error
2	1.956	1.946	−0.009
3.5	3.466	3.462	−0.003
5	4.972	4.966	−0.005
6.5	6.468	6.457	−0.010
8	7.964	7.963	−0.001
9.5	9.397	9.405	0.008
11	10.938	10.953	0.015
12	11.937	11.955	0.018

## Data Availability

Data is contained within the article, The data presented in this study are available in Experiment Results and Discussion.
